# LC/MS-Based Polar Metabolite Profiling Identified Unique Biomarker Signatures for Cervical Cancer and Cervical Intraepithelial Neoplasia Using Global and Targeted Metabolomics

**DOI:** 10.3390/cancers11040511

**Published:** 2019-04-10

**Authors:** Imran Khan, Miso Nam, Minji Kwon, Sang-soo Seo, Sunhee Jung, Ji Soo Han, Geum-Sook Hwang, Mi Kyung Kim

**Affiliations:** 1 Division of Cancer Epidemiology and Prevention, National Cancer Center, Madu-dong, Ilsandong-gu, Goyang-si, Gyeonggi-do 10408, Korea; imranbbt@ncc.re.kr (I.K.); 74433@ncc.re.kr (M.K.); 2 Integrated Metabolomics Research Group, Western Seoul Center, Korea Basic Science Institute, Seoul 03759, Korea; nms65@kbsi.re.kr (M.N.); jsh204@kbsi.re.kr (S.J.); hjs0503@kbsi.re.kr (J.S.H.); 3 Center for Uterine Cancer, National Cancer Center, Madu-dong, Ilsandong-gu, Goyang-si, Gyeonggi-do 10408, Korea; ssseomd@ncc.re.kr

**Keywords:** biomarker, cervical cancer, human papillomavirus, metabolomic analysis, metabolic pathways, plasma profiling

## Abstract

Cervical cancer remains one of the most prevalent cancers among females worldwide. Therefore, it is important to discover new biomarkers for early diagnosis of cervical intraepithelial neoplasia (CIN) and cervical cancer, preferably non-invasive ones. In the present study, we aimed to identify unique metabolic signatures for CINs and cervical cancers using global and targeted metabolomic profiling. Plasma samples (69 normal, 55 CIN1, 42 CIN2/3, and 60 cervical cancer) were examined by ultra-performance liquid chromatography-quadrupole-time-of-flight mass spectrometry (UPLC-QTOF-MS) coupled with multivariate statistical analysis. Metabolic pathways were analyzed using the integrated web-based tool MetaboAnalyst. A multivariate logistic regression analysis was conducted to evaluate the combined association of metabolites and human papillomavirus (HPV) status with the risk of cervical carcinogenesis. A total of 28 metabolites exhibiting discriminating levels among normal, CIN, and cervical cancer patients (Kruskal–Wallis test *p* < 0.05) were identified in the global profiling analysis. The pathway analysis showed significantly altered alanine, aspartate, and glutamate metabolic pathways (FDR *p*-value < 0.05) in both the discovery and validation phases. Seven metabolites (AMP, aspartate, glutamate, hypoxanthine, lactate, proline, and pyroglutamate) were discriminated between CINs and cervical cancer versus normal (area under the curve (AUC) value > 0.8). The levels of these metabolites were significantly high in patients versus normal (*p* < 0.0001) and were associated with increased risk of developing CIN2/3 and cervical cancer. Additionally, elevated levels of the seven metabolites combined with positive HPV status were correlated with substantial risk of cancer progression. These results demonstrated that metabolomics profiling is capable of distinguishing CINs and cervical cancers from normal and highlighted potential biomarkers for the early detection of cervical carcinogenesis.

## 1. Introduction

Cervical cancer is a common malignant disease in women around the world. It is the third most commonly diagnosed cancer (~485,000 cases) and the fourth leading cause of cancer-related deaths (236,000) per year worldwide [1,2]. The main cause of cervical cancer is persistent oncogenic human papillomavirus (HPV) infection. Cervical intraepithelial neoplasia (CIN) 1–3 and carcinoma in situ are the precursor lesions of cervical cancer [3]. Other factors such as sexually transmitted infections, oral contraceptive use, smoking status, parity, and diet also contribute to the development of cervical cancer [4]. Patients diagnosed with cervical cancer often show no obvious signs or symptoms at early stages, which fact can lead to misdiagnosis. The incidence of cervical cancer worldwide varies significantly due to advancements in research and precautionary measures taken by developed countries [5]. Incidence decline can be attributed to regular application of Pap tests, which can reveal CINs and carcinoma in situ before they develop into cervical cancer, and vaccination [6]. However, current diagnostics for cervical cancer are based on histological and morphological examinations, which lack sensitivity and specificity [7].

Recent developments in molecular biology, proteomics, and metabolomics have inaugurated a new era marked by the discovery of biomarkers for cervical cancer [7,8]. Metabolomic research for cervical cancer treatment applications, in particular, has attracted immense interest globally. Metabolites, the end products of several biological processes, show potential as accurate biomarkers identifying upstream biological processes such as genetic mutations and environmental changes [9,10]. Metabolomics based on platforms such as nuclear magnetic resonance (NMR) spectroscopy or mass spectrometry (MS) combined with chromatography has been utilized for comprehensive analysis and quantification of metabolites in biological systems [11,12,13]. Metabolomics as applied to cancer research has been a powerful tool for identification of metabolic changes in cancer development and progression and discovery of non-invasive biomarkers for cancer prediction, monitoring, and diagnosis [14,15,16]. Changes in metabolic profiles and pathways can provide a better understanding of dysregulated metabolism in tumor initiation and progression compared with normal (healthy) controls [17]. A well-known biomarker for cervical cancer is the squamous cell carcinoma antigen, which is elevated in 50%, 71%, and 82% of patients with stages I, II, and III–IV disease, respectively [18]. Circulating antibodies [19], CDKN3 mRNA [20], amino acids [21], and lipids [22] also have been studied as potential biomarkers for cervical cancer. However, their diagnostic accuracy and predictive performance remain uncertain.

To the best of our knowledge, this is the first study of its kind to identify, through metabolomic analysis, dysregulated metabolic pathways and potential biomarkers for CINs and cervical cancers. We performed ultra-performance liquid chromatography (UPLC)-MS-based metabolomic profiling in both CINs/cancer patients and normal controls to identify circulating biomarkers of disease progression and validate identified metabolite biomarkers. This global and targeted approach consisting of both discovery and validation steps could be helpful for discovering new biomarkers for risk prediction and early-disease diagnosis.

## 2. Results

### 2.1. General Characteristics of Study Participants

The baseline characteristics of the patients and normal (healthy) controls included in the study are listed in Table 1. The median ages calculated for the normal, CIN1, CIN2/3, and cervical cancer groups were 48, 35, 39.5, and 50 years, respectively. Among these groups, 43.5%, 54.6%, 71.4%, and 78.3% were positive for HPV infection, respectively. Significant differences (*p* < 0.05) in age, body mass index (BMI), HPV infection, marital status, education, postmenopausal status, pregnancy, and smoking status were observed among the groups; the exception was oral contraceptive use (*p* = 0.0949). Notably, the highest percentages for BMI, HPV, postmenopausal status, and positive pregnancy were recorded in the cervical cancer group.

### 2.2. Global Metabolic Profiling of Plasma by UPLC-QTOF-MS

**Figure 1:**
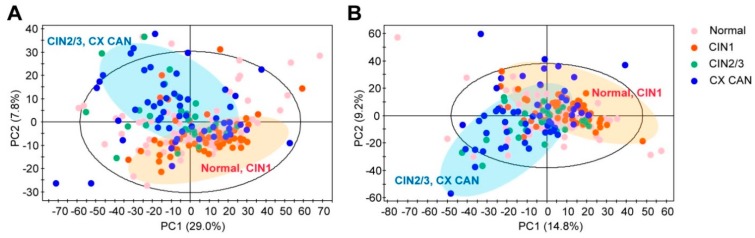
Score plots of principal component analysis (PCA) from metabolite spectra of (**A**) positive (R^2^X = 0.635, and Q^2^ = 0.472) and (**B**) negative (R^2^X = 0.666, and Q^2^ = 0.399) modes of ultra-performance liquid chromatography-quadrupole-time-of-flight mass spectrometry (UPLC-QTOF-MS). Plots of the PCA analysis reveal no clear separations of the four groups, but clustered into two groups: normal/ cervical intraepithelial neoplasia (CIN)1 group and CIN2 or 3/cervical cancer group. CIN1: Cervical intraepithelial neoplasia 1, CIN2/3: Cervical intraepithelial neoplasia 2 or 3, CX CAN: Cervical cancer.

We performed global profiling of the polar metabolites in the plasma samples to identify altered metabolites in the CIN and cervical cancer patients. During the profiling, the analytical robustness of ultra-performance liquid chromatography-quadrupole-time-of-flight mass spectrometry (UPLC-QTOF-MS) quality control (QC) samples was repeatedly analyzed. In those samples, the reproducibility of the polar metabolite features was evaluated by coefficients of variation (CV); metabolite features showing large variations were removed (CV > 20%) before conducting the statistical analysis. In the UPLC-QTOF-MS dataset, a total of 1920 metabolite features were detected in the positive mode and 2563 were detected in the negative mode. After annotation, we obtained 52 metabolite features in the positive mode and 40 in the negative mode. To determine whether the metabolic profiles (CV < 20%) of the CINs and cervical cancers were different from the normal ones, a multivariate statistical analysis using the principal component analysis (PCA) model was conducted. The score plots obtained from the PCA model are presented in Figure 1A,B. The R^2^X values of the PCA analysis were >0.6 for both the positive mode (R^2^X = 0.635) and the negative mode (R^2^X = 0.666). We excluded 26 PCA-based-outlier plasma samples (1 CIN1, 17 CIN2/3, 8 cervical cancer) from further analysis. The PCA analysis demonstrated that the four groups were not clearly discriminated; instead, the results had clustered into two groups: A normal/CIN1 group and a CIN2 or 3/cervical cancer group. Importantly, the QC samples were clustered together on the PCA score plot, which indicated the robustness of our polar profiling platform (Figure S1).

### 2.3. Differential Mapping of Metabolites in Pathway Analysis

**Figure 2:**
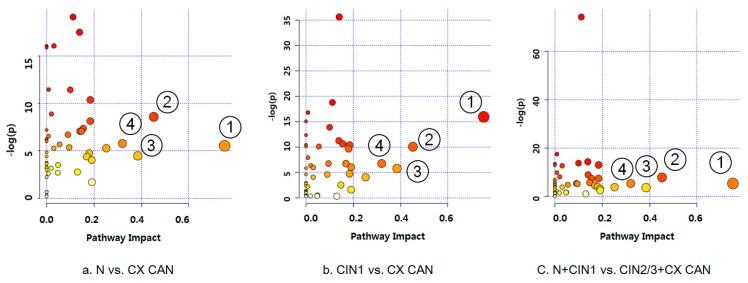
Metabolic pathway analysis in cervical carcinogenesis. Global pathway analysis was performed using MetaboAnalyst: (**a**) Normal and cervical cancer, (**b**) CIN1 and cervical cancer, (**c**) normal, CIN1, and CIN2/3, cervical cancer. ① Alanine, aspartate, and glutamate metabolism, ②Arginine and proline metabolism, ③ Taurine and hypotaurine metabolism, ④ Pyruvate metabolism. N: Normal, CIN1: Cervical intraepithelial neoplasia 1, CIN2/3: Cervical intraepithelial neoplasia 2 or 3, CX CAN: Cervical cancer.

To explore the metabolic pathways that potentially contribute to cervical cancer progression, we carried out a global metabolic pathway analysis (MetPA website: www.metaboanalyst.ca). By logarithmic transformation, totals of 37, 29, and 33 pathways from significant metabolites were obtained for normal and cervical cancer, CIN1 and cervical cancer, and normal + CIN1 and CIN2/3 + cervical cancer, respectively (false discovery rate (FDR) *p*-value < 0.05), as shown in Figure 2. Small *p*-values and large pathway-impact values indicated highly influential pathways. Based on the impact values and *p*-values, the pathways of alanine, aspartate and glutamate metabolism, arginine and proline metabolism, taurine and hypotaurine metabolism, and pyruvate metabolism were significantly altered in the cases of normal versus cervical cancer, CIN1 versus cervical cancer, and normal + CIN1 versus CIN2/3 + cervical cancer (Figure S2; FDR *p*-value < 0.05; impact value > 0.3).

### 2.4. Altered Metabolites in Patients with CINs and Cervical Cancer 

A total of 28 significantly altered metabolites were identified in the positive mode (19 metabolites) and negative mode (16 metabolites) (Kruskal–Wallis test *p* < 0.05). For global visualization of the significantly altered metabolites, hierarchical cluster analysis (HCA) and area under the curve (AUC) analysis were performed. A dendrogram produced through the HCA revealed three distinct clusters in both the positive and negative modes (Figure S3). Metabolites showing significant trends in plasma levels among the normal, CIN, and cervical cancer groups were identified as potential biomarkers for early detection of cervical cancer. Of these, we chose the top seven metabolites, namely AMP, aspartate, glutamate, hypoxanthine, lactate, proline, and pyroglutamate, on the basis of the HCA clusters and AUC analysis (Table S2).

### 2.5. Validation and Diagnostic Performance of Selected Metabolites 

**Figure 3:**
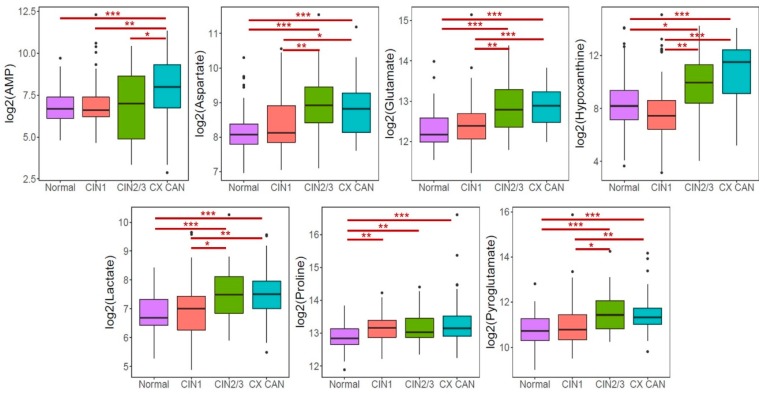
Boxplots of metabolites between normal and cervical cancer. Post-hoc multiple comparisons using Bonfferoni method was performed for significant difference. * *p* < 0.05, ** *p* < 0.01, *** *p* < 0.001. CIN1: Cervical intraepithelial neoplasia 1, CIN2/3: Cervical intraepithelial neoplasia 2 or 3, CX CAN: Cervical cancer.

The levels of the selected metabolites in a group of participants comprising 69 normal, 55 CIN1, 42 CIN2/3, and 60 cervical cancer patients (Table 1) were measured using UPLC-triple-quadrupole (TQ)-MS and analyzed in the validation phase by Bonfferoni multiple comparisons test. Figure 3 depicts the boxplots of their concentrations in the CIN, cervical cancer, and normal cases. All of the metabolites were up-regulated in CIN and cervical cancer, except for proline metabolite in the CIN1 group. Interestingly, AMP was significantly elevated in the cervical cancer group as compared to CIN and normal, whereas no significant difference was observed for proline between CIN and cervical cancer as compared to normal (*p* < 0.05).

Table 2 shows the groups’ AUC values of targeted metabolites obtained through multiple comparison analysis. The AUC values indicate the diagnostic potentials of the metabolites as unique biomarkers for identification of CINs and cervical cancers. The AUC value (0.80) of aspartate metabolite indicated a clear discrimination between the normal and CIN2/3 groups, whereas, those of glutamate and hypoxanthine metabolites clearly discriminated between normal and cervical cancer and CIN1 and cervical cancer, respectively. We thereafter combined the metabolites into various sets and subjected them to AUC analysis to evaluate their diagnostic performances as combined biomarkers for CINs and cervical cancers. The AUC value of three combined metabolites (aspartate, glutamate, and hypoxanthine) was 0.81 between the normal and CIN2/3 group and the normal and cervical cancer group; the AUC values of seven combined metabolites (AMP, aspartate, glutamate, hypoxanthine, lactate, proline, and pyroglutamate) was 0.82 between the normal and CIN2/3 group and 0.83 between the normal and cervical cancer group.

### 2.6. Association of Metabolites with CINs and Cervical Cancer Risk

Table 3 displays the associations of targeted metabolites with CIN2/3 or cervical cancer risk by multivariate logistic analysis. As can be seen, most of the metabolites were significantly associated with increased risk of developing CIN2/3 and cervical cancer from normal. Aspartate, moreover, was significantly associated with increased risk of developing cervical cancer from normal. Note also that a high level of aspartate was shown to have increased risk of developing CIN2/3 compared with CIN1 (OR, 8.92; CI, 2.38–33.4) and CIN2/3 from normal (OR, 4.31; CI, 1.38–13.5). For glutamate, the odds ratios (ORs) and correspondent confidence intervals (CIs) for the risk of developing CIN2/3 and cervical cancer from normal were 7.99 (2.31–27.6) and 6.31 (1.74–22.9), respectively. Hypoxanthine also showed an increased risk of developing CIN2/3 compared with CIN1 (OR, 7.90; CI, 2.00–31.3). The elevated level of pyroglutamate was associated with higher risks of developing cervical cancer (OR range 2.94–8.02). All of the metabolites at the elevated level showed high risks (OR range 2.12–4.49) of developing CIN2/3 + cervical cancer from normal + CIN1. These results indicated that higher levels of metabolites were significantly associated with increased risks of developing CIN2/3 and cervical cancer.

### 2.7. Combined Effects of Targeted Metabolites with HPV Status 

Table 4 displays the combined effects of HPV infection with targeted metabolites on the development risks of CIN2/3 and cervical cancer. As expected, high levels of targeted metabolites with positive HPV status showed a significant risk for development of CIN2/3 and cervical cancer. At the elevated level, proline showed the highest risk (OR 66.9; *p* for trend = 0.0001) of developing CIN2/3 from CIN1, followed by hypoxanthine, glutamate, and lactate (OR range 16.54–33.48; *p* for trend < 0.01). Similarly, proline, pyroglutamate, lactate, and glutamate at the elevated level also demonstrated increased risk for developing cervical cancer from normal (OR range 24.52–34.22; *p* for trend < 0.0009). These results indicated that the risk of developing CIN2/3 or cervical cancer is significantly higher if patients are positive for HPV infection.

### 2.8. Pathway Analysis for Quantitative Metabolites

Metabolomic pathway analysis was performed for the seven targeted metabolites using the MetPA web-based tool. The highest impact value was obtained for the alanine, aspartate, and glutamate metabolic pathways; these results were consistent with the global pathway analysis (Figures S4 and S5; impact value > 0.4, FDR *p*-value < 0.05). Metabolic pathways including arginine and proline metabolism, pyruvate metabolism, aminoacyl-tRNA biosynthesis, and D-glutamine and D-glutamate metabolism were statistically related (impact > 0.1, FDR *p*-value < 0.05).

## 3. Discussion

Alteration in metabolic pathways has been involved in the development of neoplasia and is characterized as a hallmark of cancer [23]. A growing body of evidence shows that malignant transformation requires large amounts of energy to sustain high rates of cell proliferation and energy consumption, which leads to alterations of key metabolic pathways [24,25].

In this study, we used global and targeted metabolomic profiling to identify plasma metabolite biomarkers for non-invasive early detection of cervical cancer and as risk factors for cancer development. Cervical cancer causes changes in body metabolism, and results in alterations in circulating metabolites. Thus, investigation of cervical-cancer-associated metabolic pathways can provide important insights into the different mechanisms adopted during the development and progression of tumors and proposes new methods for its early detection. Our findings showed that 28 metabolites among normal, CINs, and cervical cancers were significantly altered in the global metabolite profiling (discovery phase). These metabolites were successfully mapped to 37 normal and cervical cancer, 29 CIN1 and cervical cancer, and 33 normal + CIN1 and CIN2/3 + cervical cancer pathways. The results obtained from our pathway analysis indicated that the metabolic pathways of alanine, aspartate, and glutamate metabolism, arginine and proline metabolism, taurine and hypotaurine metabolism, and pyruvate metabolism were significantly altered between cancer patients and the normal group. In the validation phase, seven metabolites that were statistically different in cervical carcinogenesis progression were selected as potential biomarkers on the basis of AUC (>0.80) and HCA analyses. Furthermore, HPV, a known causal agent of cervical cancer development, was shown to represent high risk when combined with targeted altered metabolites in the validation phase.

Endogenous and exogenous amino acids are considered to be vital sources of nutrients that are distributed all over the body to contribute to metabolism, gene expression, cell multiplications, and inflammatory reactions. The speedy proliferation and increased metabolism of tumor cells require amino acids for protein and nucleic acids synthesis [26]. It has already been reported that amino acids such as serine, glutamine, aspartic acid, and proline metabolism play an important role in cancer progression and contribute to tumor metabolic reprogramming [27]. The analysis of sera from cancer patients often shows altered amino acid profiles compared with healthy patients [28,29]. Hasim, Aili, Maimaiti, Abudula, and Upur [21] showed that the total serum concentrations of aspartate, proline, and glutamate, among others, were gradually decreased from CIN to invasive cancer. In another study, the concentration of L-lysine essential amino acid also was decreased in cervical cancer patients [30]. Ye et al. [31] reported that metabolomics profile of serum samples obtained from cervical cancer patients exhibited reduced levels of most essential and non-essential amino acids, such as isoleucine, valine, tyrosine, and glycine, among others as compared to cervicitis and CIN. Correspondingly, we found altered amino acids profiles in cervical cancer patients compared with normal patients. By contrast, we found elevated levels of plasma aspartate, glutamate, proline, and pyroglutamate in cervical cancer patients compared with those of normal individuals (Figure 3). It is an established fact that metabolism, including that of amino acids, changes not only in cancerous cells, but also in plasma [32,33]. However, there are inconsistencies among altered amino acid profiles of cancer patients [21,34] as well as discrepancies between the reported literature and our present results. Differences among the findings of various studies can be attributed to study design, detection procedures, pre-cancerous lesion status, and sample size. However, findings similar to ours also are available. For example, some studies have reported elevated levels of amino acid profiles in cancerous patients [35,36]. Augmentation of amino acids in the plasma of cancer patients indicates the utilization of amino acids for protein synthesis required for increased cell growth [27]. These results suggest that cancer progression as characterized by the severity of invasion and metastasis is highly associated with plasma amino acid profiles [37]. However, it remains unclear how metabolic changes occurring in cancer patients influence plasma amino-acid profiles systemically.

Lactate metabolite is normally found in elevated levels in tumor hypoxia, a condition in which cells acquire energy through glycolysis instead of oxidative phosphorylation [38]. We found significantly elevated levels of lactate in CIN and cervical cancer cases compared with normal individuals. In cervical cancer patients relative to CIN2/3 patients, the lactate metabolites levels were slightly reduced. Several studies have reported similar findings of elevated levels of lactate in other types of cancer [39,40]. In contrast to our findings, Hasim et al. [41] reported reduced levels of lactate in CIN and cervical cancer as compared with normal individuals. The discrepancy in these results could have been due to differences in study design and detection methods.

Notably, we observed that metabolites from the purine metabolic pathway, AMP, and hypoxanthine were associated with cervical cancer risk. During progression from normal to CIN1, the level of hypoxanthine reduced and then increased in CIN2/3 and cervical cancer when compared to normal individuals. Hypoxanthine has been described as a protective agent against hypoxic injury and cytotoxic compounds in normal and cancerous cells [42]. It is oxidized to xanthine by xanthine oxidase (a form of xanthine oxidoreductase), whereas uric acid is the end product of their degradation [43]. Previous studies have suggested that uric acid might contribute to cancer risk, recurrence, and mortality [44], which conclusions point to the importance of this metabolic pathway in influencing cancer progression and outcomes. AMP plays important roles in the regulation of cell proliferation and in acting as an intermediate in purine metabolism. Elevated levels of AMP can be attributed to increased conversion of adenosine to AMP by adenosine kinase enzymes during cell proliferation [45]. In agreement with our findings, Satoh et al. [46] applied a multiomics approach to paired normal and tumor tissue obtained from 275 colorectal cancer patients and found that almost all metabolic genes of the de novo purine/pyrimidine synthesis pathway were up-regulated. Consistent with our findings, Sahu et al. [47] performed metabolomic analysis in urothelial carcinoma and found elevated levels of both purine and pyrimidine metabolites.

Although a cancerous tumor is limited to a certain organ, changes in metabolite levels are influenced by their metabolism in, and excretion from, several organs of the body. Therefore, the blood plasma of cancer patients contains almost all of the metabolite information related to pathogenic alterations triggered by disease, which data reveal are abnormal alterations at the gene expression and regulation levels as well as aberrations in the functions of multiple organs and tissues. However, as already noted in these pages, it remains unclear how changes in metabolic profiles occurring in pre-cancerous and cancerous patients systematically affect plasma metabolites, including amino acid.

Previous epidemiological studies have shown associations between various metabolites and cervical cancer risk; for example, levels of serum triglyceride and systolic blood pressure were positively associated with cervical cancer risk [48,49,50,51]. Walker, Burrell, Flatley, and Powers [8] performed metabolomic analysis and suggested that metabolite profiling could be used to rapidly identify women at increased risk of cervical cancer. Likewise, we also found substantial associations between targeted metabolites and CIN2/3 or cervical cancer risk (Table 3). These results indicate an increased risk of developing CIN2/3 and cervical cancer from a normal group having elevated levels of these metabolites in plasma.

Nearly all cases of cervical cancer can be attributed to HPV infection [52]. We have found 78.3% HPV positive cases for cervical cancer group (Table 1), and the results are in agreement with Lei et al. [53], who studied HPV infection in cervical cancer in a nationwide cohort 2002–2011 and found 80.6% were positive for HPV infection. In another study, Hooi et al. [54] investigated the prevalence of HPV genotypes in cervical cancers and CIN in Curaçao and found 88.5% cases were HPV positive. However, HPV status in a cervical tumor may be dependent on various factors such as type of test performed, sample quality, laboratory conditions, and sample size. HPV plays an important role in the progression of precursor lesions and cervical cancers. HPV is essential along with cofactors that aid viral persistence and progression [55]. We have discovered in our current study that HPV infection combined with elevate levels of metabolites substantially increased the risk of cancer progression. Metabolites such as aspartate, glutamate, hypoxanthine, and proline showed an increased risk for the development of CIN2/3 and cervical cancer from normal with highest risk was observed for proline (OR, 66.90; CI, 8.82–507).

Although our approach to establish a screening strategy for cervical cancer and precancerous lesions, plasma metabolomic analysis, and the search for unique metabolic pathways is novel and promising, there were some limitations to this study. First, all of the samples collected were non-fasting ones, which, in fact, potentially increased variability among our subjects. Second, the sample size was small, which could have affected the robustness of our statistical analysis. Third, we performed UPLC-QTOF-MS analysis, which might not be an appropriate screening tool for large populations, due to its high cost in developing countries. Fourth, personal and demographic factors such as sex, age, BMI, diet, and smoking status might influence the metabolomic landscape. Further study is needed to optimize and validate cost-effective methods for detection of plasma metabolic biomarkers. Additionally, careful matching of known risk factors and adjustment of covariates in statistical analysis could be considered in order to avoid false positivity. Nevertheless, global profiling of plasma metabolites with targeted validation and comprehensive collection of epidemiological information within a proper hospital setup could offer important resources for identifying unique metabolic biomarkers suitable for clinical application.

## 4. Materials and Methods

### 4.1. Study Population and Sample Collection

This study included subjects aged 18 to 65 years who had participated in the Korean Prospective Study of the Transition of Human Papillomavirus into Cervical Carcinoma since 2006. These women were randomly selected from the six gynecology and oncology departments located in South Korean university hospitals. Details on the study’s design criteria are available in our previous paper [56]. All of the patients had been diagnosed with CINs or cervical cancers histologically. Blood samples were collected from the participants in a non-fasting state and centrifuged at 3000 rpm for 20 min at 4 °C; the obtained plasma was stored at −80 °C until further analysis. A total of 205 samples (70 normal, 54 CIN1, 27 CIN2/3, 54 cervical cancer) were selected for positive-mode global analysis, and 202 (67 normal, 54 CIN1, 27 CIN2/3, 54 cervical cancer) for negative-mode global analysis. Additionally, a total of 226 plasma samples (69 normal, 55 CIN1, 42 CIN2/3, 60 cervical cancer) were selected for targeted analysis. According to the requirements of the National Cancer Center (NCC)’s Institutional Review Board, written consent was obtained from all of the participants. This study was approved by the NCC’s Institutional Ethics Committee (IRB No. NCC2016-0147).

### 4.2. Global Metabolite Profiling Using UPLC-QTOF-MS

A UPLC-QTOF-MS-based platform was used to analyze polar metabolites from chloroform/methanol extract. Briefly, plasma samples (50 μL) were extracted using a 500 μL chloroform:methanol (2:1, v/v) solution and 100 μL water. The aqueous supernatant was vacuum dried and reconstituted into 250 μL water:acetonitrile (4:1, v/v) solution. Finally, 5 μL solution was injected into the UPLC-QTOF-MS system. The UPLC-QTOF-MS analysis was performed using the ACQUITY UPLC system (Waters, Milford, MA, USA) coupled with a triple TOF™ 5600 mass spectrometer equipped with an electrospray ionization (ESI) source (AB Sciex, Concord, ON, Canada). Chromatographic separation was carried out at 40 °C on an Acquity UPLC HSS T3 column (2.1 mm × 100 mm, 1.7 μm; Waters) with a binary gradient at a flow rate of 0.4 mL/min. The mobile phases consisted of 0.1% formic acid in water (solvent A) and 0.1% formic acid in acetonitrile (solvent B). The steps of the gradient profile used to equilibrate the initial gradient for subsequent runs were as follows: 1% B, 1%–10% B from 0–3 min, 10%–30% B from 3–5 min, 30%–50% B from 5–10 min, 50%–70% B from 10–13 min, 70%–90% B from 13–15 min, 90%–1% B from 15–18 min, 1% B from 18–20 min.

The mass spectrometer was operated in both the positive and negative ion modes, and data were acquired in the 50–1000 m/z mass range. Total ion chromatograms were acquired according to the following operation parameters: Capillary voltages of +5500 V and −4500 V for the positive and negative modes, a nebulizer pressure of 60 psi, a drying gas pressure of 60 psi, a curtain gas pressure of 30 psi, a source temperature of 500 °C, a declustering potential of ± 90 eV, a collision energy of ± 10 eV for single MS, and a collision energy of ± 35 eV for MS/MS. Data from the MS/MS analyses were acquired by automatic fragmentation, in which process the five most intense mass peaks were fragmented. Mass accuracy was maintained by use of an automated calibrant delivery system interfaced with the second inlet of the DuoSpray source. Equal amounts of all of the samples were pooled to generate QC samples, which were analyzed prior to sample acquisition and after every 15 samples in order to monitor the stability and reproducibility of the analytical system.

### 4.3. Metabolite Quantification Using UPLC-TQ-MS

Modern TQ-MS provides the ability to detect and quantify a large number of metabolites. The present quantification of targeted metabolites was performed on Agilent 1290 Infinity II LC and Agilent 6495 Triple Quadrupole MS systems equipped with an Agilent Jet Stream ESI source (Agilent Technologies, Palo Alto, CA, USA). MassHunter Workstation (Ver B.06.00, Agilent Technologies) software was used for data acquisition and analysis. Chromatographic separation was performed using a Scherzo SM-C18 column (2 mm × 100 mm, 3 μm; Imtakt, Kyoto, Japan). The flow rate and injection volume were set at 0.35 mL/min and 1 μL, respectively. Mobile phase A consisted of 0.1% formic acid in water, and phase B consisted of 0.1% formic acid in methanol. The linear gradient used for elution and equilibration of the initial gradient for subsequent runs was as follows: 5% B from 0–3 min, 5–90% B from 3–10 min, 90% B from 10–12 min, 5–95% B from 12–13 min, 5% B from 13–15 min. Quantification was performed in the multiple reaction monitoring (MRM) mode, and the optimal conditions for each metabolite were determined by flow injection of individual standards (100 ng/mL in 20% methanol) into the ESI source in the positive and negative ion modes. ^13^C_6_-Phenylalanine was used as an internal standard. The compound retention times and MRM transitions are summarized in Table S1.

### 4.4. Statistical Analysis

**Figure 4:**
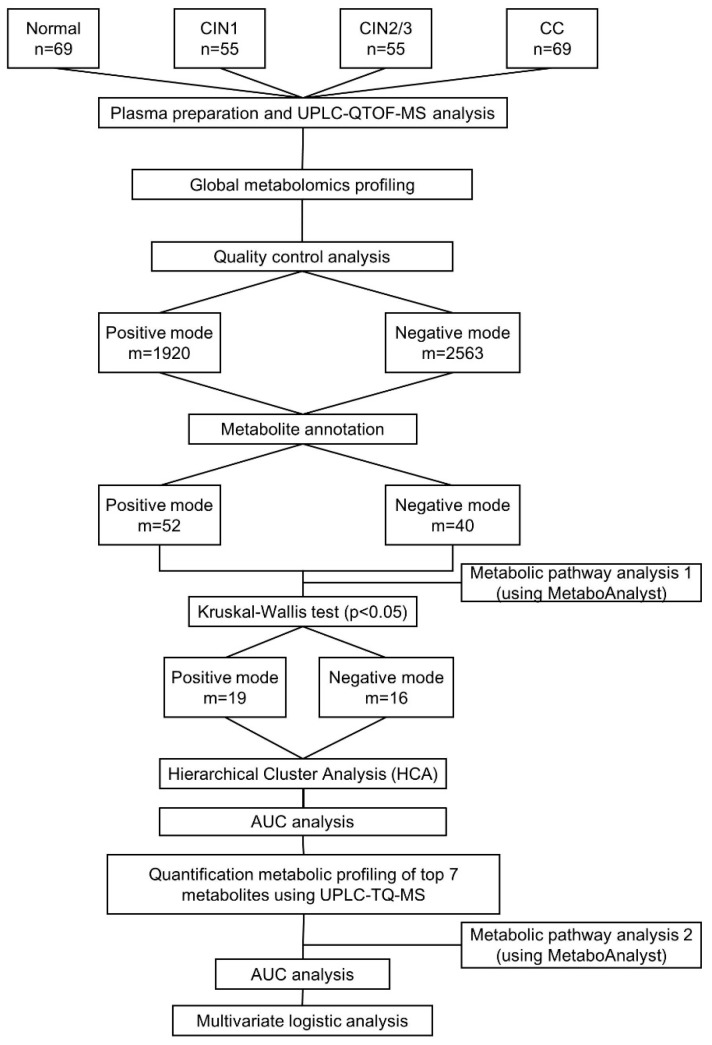
Schematic flow chart of metabolomic analysis in present study. UPLC-QTOF-MS: Ultra-performance liquid chromatography-quadrupole time-of-flight mass spectrometry; UPLC-TQ-MS: Ultra-performance lipid chromatography-triple quadruple mass spectrometry; AUC: Area under the curve; HCA: Hierarchical cluster analysis.

A multivariate statistical analysis was performed using SIMCA-P+ version 12.0 (Umetrics, Umeå, Sweden). PCA using an unsupervised method was applied to obtain an overview of the metabolic data. All of the metabolite variables obtained from the UPLC-QTOF-MS datasets were scaled to unit-variances prior to conducting the PCA. The model validity was evaluated from model parameters *R^2^* and *Q^2^*, which provide information for the interpretability and predictability, respectively, of the model. The differences between groups were compared using Kruskal–Wallis test for continuous variables and chi-square test for categorical variables. The metabolites obtained from global analysis were subjected to Kruskal–Wallis analysis, HCA, and AUC analysis [57,58]. Afterwards, seven metabolites were selected on the basis of the HCA and AUC values. Post-hoc multiple comparisons using the Bonfferoni method were applied to identify significant inter-group differences in the targeted metabolites [59]. An AUC analysis also was performed on the targeted metabolites. Metabolite enrichment was log transformed before being subjected to pathway analysis in MetaboAnalyst version 4.0 (Xia Lab, McGill University, Montreal, QC, Canada) [60]. Unconditional logistic regression was conducted to estimate the ORs and the correspondent CIs (95%), which were adjusted for age, BMI, marital status, education, menopause status, pregnancy, and smoking status. To categorize the metabolites, the median value in the normal group was calculated and used as the cutoff point for dichotomization into high- and low-risk groups. The combined effects of HPV status with metabolites were calculated using unconditional logistic regression analysis, and the estimated ORs and CIs (95%) after the variables were log2-transformed. All of the statistical tests were 2-sided, with the significance set at *p* < 0.05. All of the other statistical analyses and visualizations were performed using the ggplot2 packages in the R platform [58,61]. The overall scheme of the metabolomic analysis for the discovery of novel biomarkers for cervical carcinogenesis is presented in Figure 4.

## 5. Conclusions

The use of metabolomics for detection of cervical cancer biomarkers has been shown by previous studies as well as the present one to be quite promising. The results obtained in the current study showed that 28 metabolites were significantly altered among normal controls, CINs, and cervical cancers in the global profiling phase. We determined that analysis of circulating AMP, aspartate, glutamate, hypoxanthine, lactate, proline, and pyroglutamate levels have the potential to distinguish patients with CINs and cervical cancer from normal (healthy) patients. The risk of developing cervical cancer was high in cases where the metabolite levels were elevated. The risk of developing precancerous lesions and invasive cancer was even higher where patients had elevated levels of metabolites and showed positivity for HPV infection. Further studies are necessary in order to fully understand the association of metabolic changes with cervical cancer progression and their diagnostic potentials.

## Figures and Tables

**Table 1:** General characteristics of study subjects.

Variables		Normal, *n* = 69	CIN1, *n* = 55	CIN2/3, *n* = 42	CX CAN, *n* = 60	*p* ^a^
Age (years)		48 (43, 51)	35 (31, 40)	39.5 (33, 49)	50 (42, 51)	<0.0001
BMI (Kg/m^2^)		21.64 (20.5, 23.2)	20.6 (19.4, 21.9)	20.8 (19.8, 23.4)	23.2 (20.6, 25.7)	0.0003
HPV	Positive	30 (43.5)	30 (54.6)	30 (71.4)	47 (78.3)	0.0002
Marital status	Single	4 (5.8)	21 (38.2)	7 (20)	4 (6.67)	<0.0001
	Married	59 (85.5)	29 (52.7)	24 (68.6)	47 (78.3)	
	Divorce, bereavement	6 (8.7)	5 (9.1)	4 (11.4)	9 (15)	
Education	≤9 y	12 (17.4)	2 (3.6)	6 (17.1)	30 (50)	<0.0001
	9–12 y	24 (34.8)	19 (34.6)	13 (37.1)	21 (35)	
	≥12 y	33 (47.8)	34 (61.8)	16 (45.7)	9 (15.0)	
Postmenopausal	Yes	28 (40.6)	4 (7.3)	8 (22.9)	34 (56.7)	<0.0001
Pregnancy	No	4 (5.8)	21 (38.2)	9 (25.7)	5 (8.3)	<0.0001
	Yes	65 (94.2)	34 (61.8)	26 (74.3)	55 (91.7)	
Oral contraceptive	Yes	11 (15.9)	8 (14.8)	12 (34.3)	9 (17.7)	0.0949
Smoking status	Yes, include past	8 (11.8)	18 (32.7)	7 (20)	7 (11.7)	0.0097

Data are present as median (25%, 75%), number (%). CIN1: cervical intraepithelial neoplasia 1, CIN2/3: cervical intraepithelial neoplasia 2 or 3, CX CAN: cervical cancer. ^a^ Kruskal–Wallis test in continuous variables and chi-square test in categorical data.

**Table 2:** Area under the curves (AUCs) among normal, CINs, and cervical cancers.

Metabolite	N vs. CIN2/3	N vs. CX CAN	CIN1 vs. CIN2/3	CIN1 vs. CX CAN	N+CIN1 vs. CIN2/3 + CX CAN
AMP	0.53	0.71	<0.50	0.68	0.62
Aspartate	0.80	0.76	0.71	0.67	0.74
Glutamate	0.76	0.81	0.69	0.73	0.76
Hypoxanthine	0.68	0.77	0.72	0.80	0.74
Lactate	0.74	0.74	0.67	0.69	0.71
Proline	0.68	0.71	0.51	0.54	0.62
Pyroglutamate	0.72	0.74	0.68	0.69	0.71
Aspartate,Glutamate	0.80	0.81	0.70	0.71	0.76
Aspartate,Hypoxanthine	0.82	0.76	0.73	0.73	0.73
Glutamate,Hypoxanthine	0.76	0.81	0.67	0.76	0.76
Aspartate,Glutamate,Hypoxanthine	0.81	0.81	0.73	0.74	0.75
AMP,Aspartate,Glutamate,Hypoxanthine	0.82	0.82	0.71	0.73	0.75
Aspartate,Glutamate,Hypoxanthine,Lactate	0.80	0.80	0.73	0.73	0.76
Aspartate,Glutamate,Hypoxanthine,Proline	0.82	0.81	0.72	0.72	0.75
Aspartate,Glutamate,Hypoxanthine,Pyroglutamate	0.80	0.81	0.74	0.76	0.76
AMP,Aspartate,Glutamate,Hypoxanthine,Lactate	0.81	0.82	0.71	0.73	0.75
AMP,Aspartate,Glutamate,Hypoxanthine,Proline	0.82	0.83	0.72	0.72	0.75
AMP,Aspartate,Glutamate,Hypoxanthine,Pyroglutamate	0.81	0.82	0.73	0.77	0.78
Aspartate,Glutamate,Hypoxanthine,Lactate,Proline	0.82	0.81	0.72	0.72	0.75
Aspartate,Glutamate,Hypoxanthine,Lactate,Pyroglutamate	0.81	0.80	0.74	0.76	0.77
AMP,Aspartate,Glutamate,Hypoxanthine,Lactate,Proline	0.82	0.83	0.71	0.72	0.75
AMP,Aspartate,Glutamate,Hypoxanthine,Lactate,Pyroglutamate	0.81	0.82	0.72	0.78	0.78
Aspartate,Glutamate,Hypoxanthine,Lactate,Proline,Pyroglutamate	0.82	0.82	0.73	0.77	0.78
AMP,Aspartate,Glutamate,Hypoxanthine,Lactate,Proline,Pyroglutamate	0.82	0.83	0.72	0.78	0.78

AMP: Adenosine monophosphate, N: Normal, CIN1: Cervical intraepithelial neoplasia 1, CIN2/3: Cervical intraepithelial neoplasia 2 or 3, CX CAN: Cervical cancer.

**Table 3:** Associations of targeted metabolites with CIN2/3 and cervical cancer risk.

Metabolite	N	CIN2/3	N vs. CIN2/3	CX CAN	N vs. CX CAN	CIN1	CIN23	CIN1 vs. CIN2/3	CX CAN	CIN1 vs. CX CAN	N + CIN1	CIN23 + CX CAN	N + CIN1 vs. CIN23 + CX CAN
	*n* = 69	*n* = 42	mOR (95% CI)	*n* = 60	mOR (95% CI)	*n* = 55	*n* = 42	mOR (95% CI)	*n* = 60	mOR (95% CI)	*n* = 124	*n* = 102	mOR (95% CI)
AMP													
Low	49.3	38.1	ref	25	ref	40.1	35.7	ref	25	ref	50	30.4	ref
High	50.7	61.9	2.11 (0.79–5.63)	75	1.52 (0.54–4.28)	50.9	64.3	4.34 (1.29–14.6)	75	0.62 (0.12–3.28)	50	69.6	2.34 (1.13–4.83)
Aspartate													
Low	50.7	11.9	ref	21.7	ref	49.1	14.3	ref	25	ref	50	20.6	ref
High	49.3	88.1	4.31 (1.38–13.5)	78.3	1.64 (0.59–4.58)	50.9	85.7	8.92 (2.38–33.4)	75	0.81 (0.15–4.24)	50	79.4	2.80 (1.36–5.78)
Glutamate													
Low	50.7	11.9	ref	8.3	ref	50.9	28.6	ref	20	ref	50.8	14.7	ref
High	49.3	88.1	7.99 (2.31–27.6)	91.7	6.31 (1.74–22.9)	49.1	71.4	2.68 (0.87–8.20)	80	0.60 (0.10–3.71)	49.2	85.3	4.49 (2.05–9.82)
Hypoxanthine													
Low	49.3	23.8	ref	15	ref	50.9	14.3	ref	8.3	ref	50.8	17.7	ref
High	50.7	76.2	5.30 (1.64–17.1)	85	4.33 (1.30–14.5)	49.1	85.7	7.90 (2.00–31.3)	91.7	2.36 (0.42–13.3)	49.2	32.3	4.26 (1.95–9.28)
Lactate													
Low	50.7	14.3	ref	13.3	ref	50.9	33.3	ref	25	ref	50	20.6	ref
High	49.3	85.7	5.18 (1.74–15.4)	86.7	3.93 (1.24–12.5)	49.1	66.7	1.98 (0.68–5.78)	75	0.96 (0.17–5.50)	50	79.4	2.30 (1.13–4.69)
Proline													
Low	50.7	23.8	ref	20	ref	50.9	54.8	ref	53.3	ref	50	30.4	ref
High	49.3	76.2	5.13 (1.71–15.4)	80	5.23 (1.50–18.3)	49.1	45.2	1.04 (0.35–3.09)	46.7	0.69 (0.12–3.94)	50	69.6	2.12 (1.02–4.43)
Pyroglutamate													
Low	50.7	23.8	ref	10	ref	49.1	23.8	ref	11.7	ref	50.8	16.7	ref
High	49.3	76.2	2.94 (1.10–7.84)	90	8.02 (2.13–30.3)	50.9	76.2	5.46 (1.63–18.4)	88.3	4.54 (0.65–32.0)	49.2	83.3	4.14 (1.92–8.92)

mOR: Multivariate odds ratio, CI: Confidence interval, ref: Reference, AMP: Adenosine monophosphate, N: Normal, CIN1: Cervical intraepithelial neoplasia 1, CIN2/3: Cervical intraepithelial neoplasia 2 or 3, CX CAN: Cervical cancer. Adjusted for age, BMI, marital status, education, menopause status, parity, smoking status.

**Table 4:** Combined effects of metabolites with human papillomavirus (HPV) status on CIN2/3 and cervical cancer risk.

Metabolite	HPV	N vs. CIN2/3	N vs. CX CAN	CIN1 vs. CIN2/3	CIN1 vs. CX CAN	N+CIN1 vs. CIN2/3 + CX CAN
		mOR (95% CI)	mOR (95% CI)	mOR (95% CI)	mOR (95% CI)	mOR (95% CI)
AMP						
Low	Neg	ref	ref	ref	ref	ref
Low	Pos	6.56 (0.99–43.2)	5.36 (0.99–29.0)	5.32 (0.79–35.9)	5.31 (0.98–28.8)	3.36 (1.02–11.1)
High	Neg	1.90 (0.27–13.3)	0.87 (0.12–6.06)	1.82 (0.26–12.8)	0.85 (0.12–5.94)	1.24 (0.32–4.82)
High	Pos	12.19 (2.21–67.1)	8.54 (1.63–44.8)	12.78 (2.31–70.7)	8.46 (1.61–44.6)	9.33 (2.94–29.6)
*p* interaction ^1^		0.0022	0.0184	0.0008	0.0184	<0.0001
*p* trend ^2^		0.0065	0.0489	0.0034	0.0513	0.0002
Aspartate						
Low	Neg	ref	ref	ref	ref	ref
Low	Pos	2.52 (0.31–20.5)	3.58 (0.67–19.2)	3.45 (0.47–25.3)	4.67 (0.93–23.4)	2.47 (0.73–8.34)
High	Neg	1.64 (0.24–11.2)	0.46 (0.06–3.46)	1.74 (0.25–12.0)	0.50 (0.07–3.76)	1.08 (0.28–4.13)
High	Pos	13.93 (2.62–74.0)	7.71 (1.60–37.1)	13.52 (2.55–71.7)	6.97 (1.45–33.4)	9.15 (2.96–28.3)
*p* interaction		<0.0001	0.0067	0.0002	0.0236	<0.0001
*p* trend		0.0009	0.041	0.0013	0.0802	<0.0001
Glutamate						
Low	Neg	ref	ref	ref	ref	ref
Low	Pos	0.98 (0.11–9.18)	1.14 (0.11–12.3)	3.62 (0.69–18.9)	3.28 (0.60–18.0)	2.76 (0.68–11.3)
High	Neg	1.49 (0.20–11.3)	1.33 (0.16–10.7)	1.51 (0.21–10.6)	0.92 (0.12–7.06)	2.29 (0.56–9.40)
High	Pos	27.18 (4.23–175)	24.52 (3.61–167)	17.01 (3.38–85.6)	14.33 (2.64–77.8)	14.85 (4.27–51.7)
*p* interaction		<0.0001	<0.0001	0.0002	0.001	<0.0001
*p* trend		<0.0001	0.0005	0.0006	0.0047	<0.0001
Hypoxanthine						
Low	Neg	ref	ref	ref	ref	ref
Low	Pos	7.59 (0.93–61.9)	3.35 (0.45–24.9)	46.45 (2.11–999)	10.83 (0.73–161)	2.11 (0.56–8.04)
High	Neg	5.25 (0.61–45.3)	1.82 (0.24–13.6)	9.76 (0.70–135)	1.27 (0.18–9.21)	1.58 (0.42–6.05)
High	Pos	21.89 (3.36–142)	14.88 (2.57–86.2)	33.48 (2.83–396)	8.19 (1.44–46.6)	11.18 (3.55–35.2)
*p* interaction		0.0005	0.0009	0.003	0.0076	<0.0001
*p* trend		0.0006	0.0023	0.0024	0.0216	<0.0001
Lactate						
Low	Neg	ref	ref	ref	ref	ref
Low	Pos	2.33 (0.31–17.8)	5.35 (0.69–41.7)	10.40 (1.80–60.0)	3.30 (0.71–15.4)	4.24 (1.23–14.6)
High	Neg	1.86 (0.27–12.8)	2.79 (0.36–21.4)	3.57 (0.50–25.3)	0.34 (0.03–3.8)	1.63 (0.42–6.36)
High	Pos	17.99 (3.31–97.8)	26.84 (3.69–196)	16.54 (2.86–95.7)	12.01 (2.35–61.3)	9.93 (3.05–32.4)
*p* interaction		<0.0001	0.0006	0.0105	0.0022	<0.0001
*p* trend		0.0002	0.0013	0.0058	0.0139	0.0003
Proline ^3^						
Low	Neg	ref	ref	ref	ref	ref
Low	Pos	–	4.16 (0.52–33.5)	6.61 (1.53–28.6)	4.83 (1.07–21.8)	7.65 (1.95–29.9)
High	Neg	–	2.79 (0.35–22.2)	4.29 (0.63–29.2)	1.38 (0.18–10.7)	3.10 (0.73–13.2)
High	Pos	-	34.22 (3.96–296)	66.90 (8.82–507)	22.04 (3.60–135)	13.46 (3.51–51.7)
*p* interaction		-	0.0003	0.0004	0.0028	<.0001
*p* trend		-	0.0009	0.0001	0.0029	<.0001
Pyroglutamate						
Low	Neg	ref	ref	ref	ref	ref
Low	Pos	6.76 (0.92–49.9)	3.81 (0.39–37.7)	6.90 (0.94–50.5)	6.71 (0.75–60.2)	4.23 (1.07–16.8)
High	Neg	2.68 (0.34–21.0)	4.59 (0.56–37.4)	2.83 (0.36–22.0)	4.76 (0.60–37.7)	3.15 (0.77–12.9)
High	Pos	15.50 (2.79–86.0)	27.93 (4.03–194)	15.79 (2.86–87.1)	22.76 (3.47–149)	16.17 (4.55–57.5)
*p* interaction		0.001	0.0004	0.001	0.0013	<0.0001
*p* trend		0.0019	0.0005	0.0017	0.0011	<0.0001

CI: Confidence interval; AMP: Adenosine monophosphate; N: Normal, CIN1: Cervical intraepithelial neoplasia 1, CIN2/3: Cervical intraepithelial neoplasia 2 or 3, CX CAN: Cervical cancer, POS: Positive; NEG: Negative. Adjusted for age, BMI, marital status, education, menopause status, parity, smoking status. ^1^ P is for the interaction provided by the logistic regression for multiplicative terms (a × b). ^2^ P is for the trend according to the order of combination provided by the logistic regression analysis. ^3^ The analysis of proline in N vs. CIN2/3 groups was not possible because of the small sample size.

## Supplementary Materials

The following are available online at https://www.mdpi.com/2072-6694/11/4/511/s1, Figure S1: PCA score plots with QC clusters, Figure S2: Construction of the altered metabolism pathways using MetPA, Figure S3: HCA results (a. positive, b. negative mode), Figure S4: Construction of altered metabolic pathways using MetPA analysis for targeted metabolites, Figure S5: Alanine, aspartate, and glutamate metabolism (C00049: Asparate, C00025: Glutamate). Table S1: Retention times and multiple reaction monitoring transitions of plasma metabolites quantified by UPLC-TQ-MS, Table S2: AUC values of significantly altered metabolites (*p* value < 0.05) in given HCA clusters.

## Abbreviations

AMP	Adenosine monophosphate
AUC	Area under the curve
BMI	Body mass index
CI	Confidence interval
CIN	Cervical intraepithelial neoplasia
CV	Coefficients of variation
FDR	False discovery rate
HCA	Hierarchical cluster analysis
HPV	Human papillomavirus
OR	Odds ratio
PCA	Principal component analysis
QC	Quality control
UPLC-QTOF-MS	Ultra-performance liquid chromatography-quadrupole-time-of-flight mass spectrometry
UPLC-TQ-MS	Ultra-performance liquid chromatography-triple-quadrupole mass spectrometry
