# Supplementary material for: LC/MS-Based Polar Metabolite Profiling Identified Unique Biomarker Signatures for Cervical Cancer and Cervical Intraepithelial Neoplasia Using Global and Targeted Metabolomics
Source: Cancers (Basel). 2019 Apr 10;11(4):511. doi: 10.3390/cancers11040511 (PMC6521312; doi:10.3390/cancers11040511)
Supplement: Supplementary file 1 [file cancers-11-00511-s001.pdf]

Supplementary Materials: LC/MS-Based Polar Metabolite Profiling Identified Unique Biomarker Signatures for Cervical Cancer and Cervical Intraepithelial Neoplasia Using Global and Targeted Metabolomics

Imran Khan, Miso Nam, Minji Kwon, Sang-soo Seo, Sunhee Jung, Ji Soo Han, Geum-Sook Hwang and Mi Kyung Kim

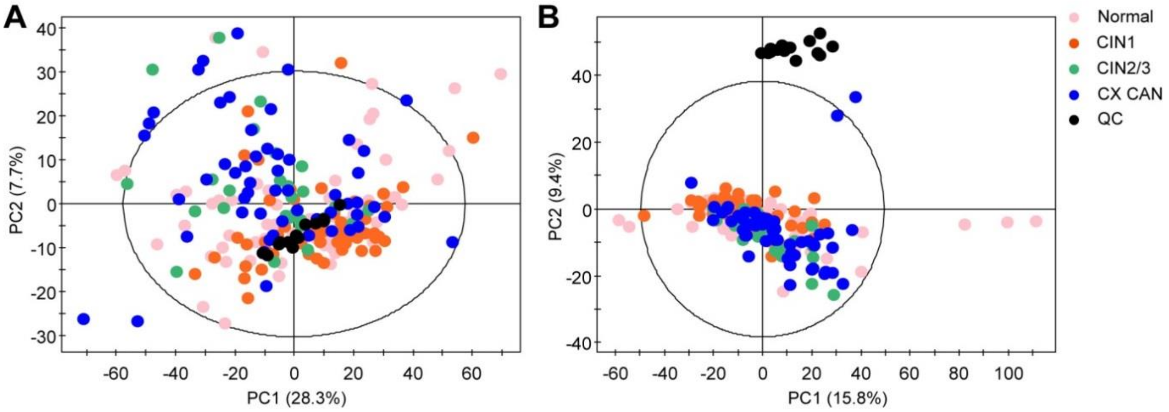

Figure S1. PCA score plots with quality control (QC) cluster. Plots show spectra of metabolites in (**A**) positive (R^2X = 0.652, and Q^2 = 0.488) and (**B**) negative (R^2X = 0.699, and Q^2 = 0.482) modes of UPLC-QTOF-MS. CIN1: cervical intraepithelial neoplasia 1, CIN2/3: cervical intraepithelial neoplasia 2 or 3, CX CAN: cervical cancer.

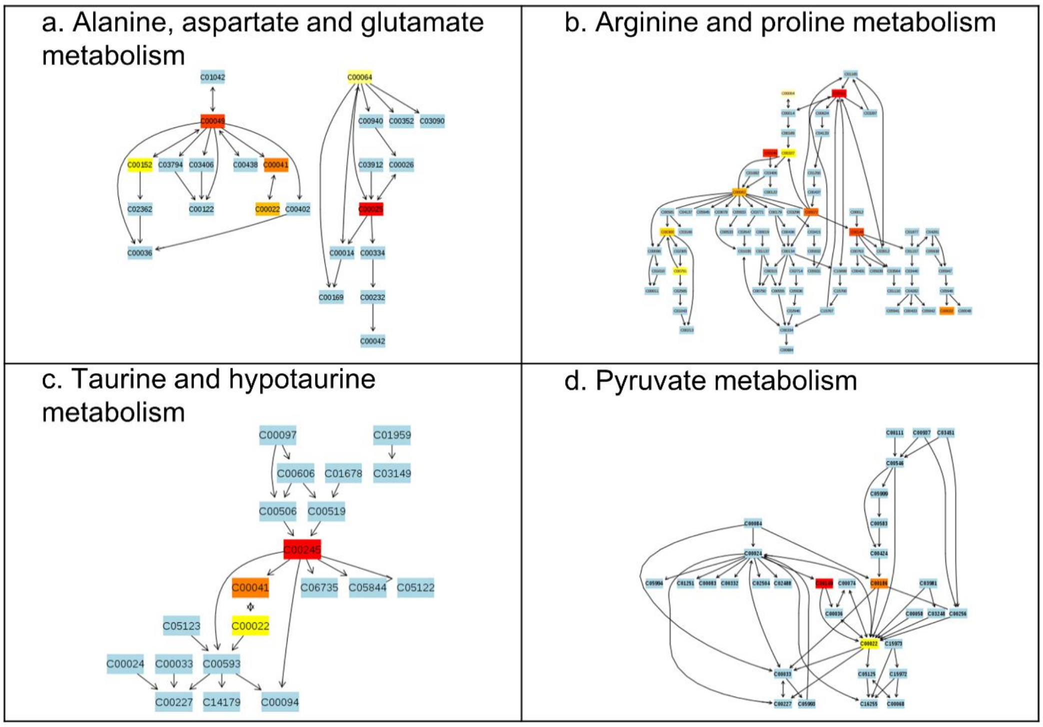

Figure S2. Construction of the altered metabolism pathways using MetPA. (**a**) Alanine, aspartate and glutamate metabolism (colored metabolites C00049: aspartate, C00152: asparagine, C00041: alanine, C00022: pyruvate, C00064: glutamine, C00025: glutamate); (**b**) Arginine and proline metabolism (colored metabolotes: C00300: creatine, C00791: creatinine, C00062: arginine, C00049: aspartate, C00064:

glutamine, C00327: citrulline, C00077: ornithine, C00025: glutamate, C00148: proline, C00022: pyruvate); (**c**) Taurine and hypotaurine metabolism (colored metabolites C00245: taurine, C00041: alanine, C00022: pyruvate); (**d**) Pyruvate metabolism (colored metabolites C00149: malate, C00186: lactate, C00022: pyruvate). Colors of the metabolites from yellow to red represent different level of significance, whereas light blue color represents that these metabolites are not in data set and are used as background for enrichment analysis.

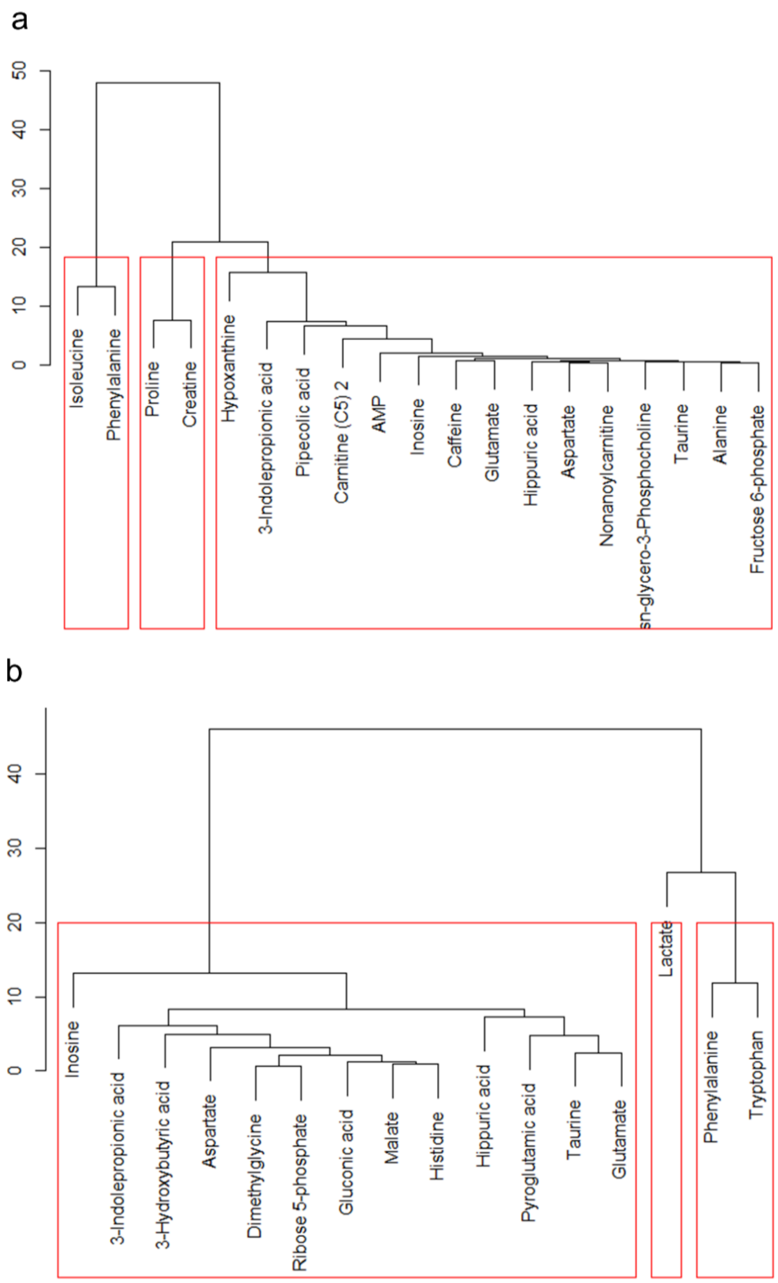

Figure S3. Results of hierarchical cluster analysis (**a**. positive, **b**. negative mode). The red squares represent clusters.

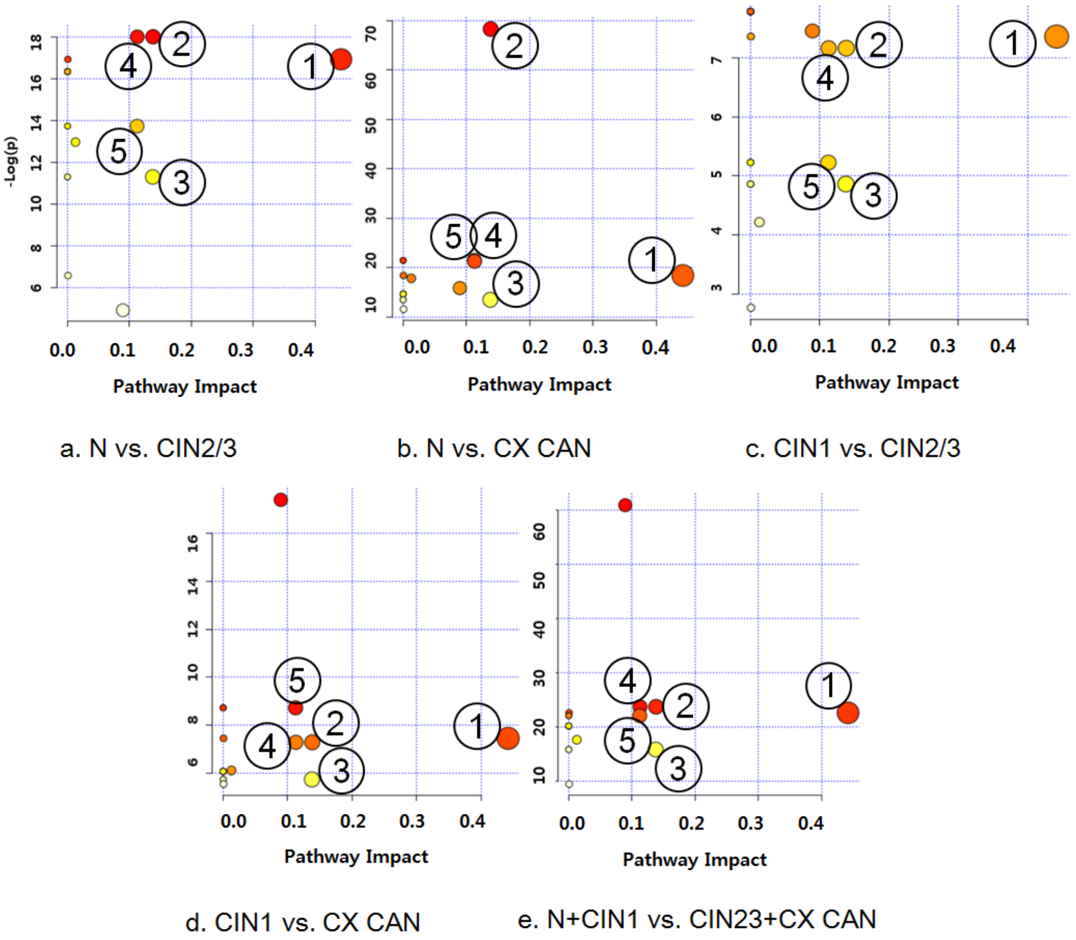

Figure S4. Construction of altered metabolic pathways using MetPA analysis for targeted metabolites: (**a**) normal and CIN2/3, (**b**) normal and cervical cancer, (**c**) CIN1 and CIN2/3, (**d**) CIN1 and cervical cancer, (**e**) normal, CIN1 and CIN2/3, cervical cancer. ① Alanine, aspartate and glutamate metabolism, ② arginine and proline metabolism, ③ pyruvate metabolism, ④ aminoacyl-tRNA biosynthesis, ⑤ D-Glutamine and D-glutamate metabolism, N: normal, CIN1: cervical intraepithelial neoplasia 1, CIN2/3: cervical intraepithelial neoplasia 2 or 3, CX CAN: cervical cancer.

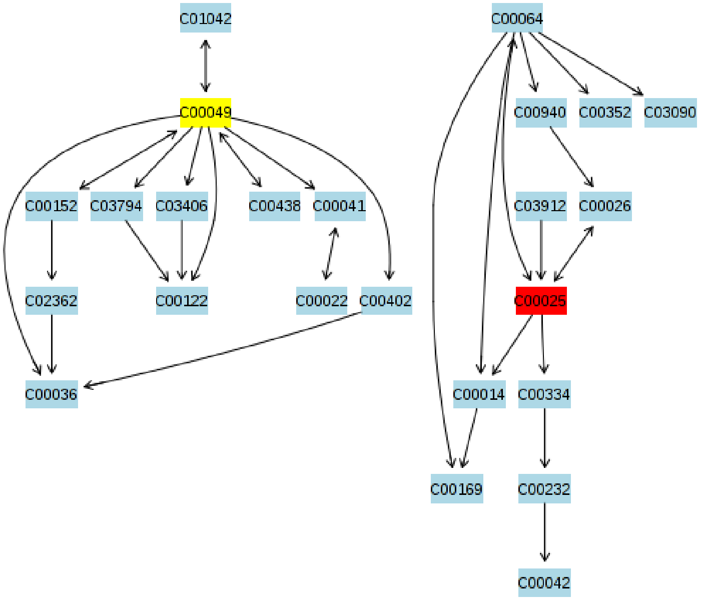

Figure S5. Alanine, aspartate and glutamate metabolism (C00049: asparate, C00025: glutamate). Colors of the metabolites from yellow to red represent different level of significance, whereas light blue color represents that these metabolites are not in data set and are used as background for enrichment analysis.

Table S1. Retention times and multiple reaction monitoring transitions of plasma metabolites quantified by UPLC-TQ-MS.

Compound	Retention time (min)	Ionization Mode	Precursor ion (*m*/*z*)	Collision energy (eV)	MRM ion transitions (*m*/*z*)
AMP	3.02	+	348	20	136
Aspartate	0.8	+	134	14	74
Glutamate	0.74	+	148	16	84
Hypoxanthine	1.49	+	137	20	110
Lactate	1.32	-	89	1	89
Proline	0.75	+	116	16	70
Pyroglutamate	2.16	+	130	12	84

AMP: Adenosine monophosphate, MRM: multiple reaction monitoring.

Table S2. AUC values of significantly altered metabolites (p value < 0.05) in given HCA clusters.

Metabolites	Cluster	p [a]	AUC				
			N vs. CX CAN	N vs. CIN2/3	CIN1 vs. CIN2/3	CIN1 vs. CX CAN	N+CIN1 vs. CIN2/3+CX CAN
Positive mode (n = 19)							
**Phenylalanine**	1	0.00673	0.663756614	0.532275132	0.582304527	0.562757202	0.566974823
Isoleucine	1	0.0283	0.65	0.621164021	0.514403292	0.563100137	0.592348544
**Proline**	2	0.01357	0.652380952	0.602116402	0.530864198	0.542866941	0.577399685
Creatine	2	0.01255	0.612962963	0.685185185	0.670781893	0.596021948	0.622344611
**Hypoxanthine**	3	<0.0001	0.806878307	0.594708995	0.667352538	0.836762689	0.746164437
**AMP**	3	<0.0001	0.795238095	0.711640212	0.7050754	0.7750343	0.7512785
**Glutamate**	3	<0.0001	0.774338624	0.592063492	0.579561043	0.75308642	0.696990559
sn-glycero-3-Phosphocholine	3	<0.0001	0.753703704	0.677777778	0.3600823	0.7098765	0.7015146
Aspartate	3	<0.0001	0.742857143	0.555026455	0.606310014	0.785322359	0.691483084
Nonanoylcarnitine	3	<0.0001	0.730952381	0.552380952	0.4876543	0.7184499	0.6661094
Inosine	3	<0.0001	0.721693122	0.607407407	0.3607682	0.7451989	0.6865657
Caffeine	3	0.00139	0.693121693	0.604232804	0.549382716	0.659807956	0.650177026
Pipecolic acid	3	0.00413	0.678571429	0.619047619	0.425925926	0.62037037	0.639752164
Taurine	3	0.00176	0.652645503	0.615873016	0.655006859	0.686213992	0.64781668
Alanine	3	0.04372	0.643650794	0.50952381	0.524005487	0.607681756	0.576514555
Hippuric acid	3	0.02244	0.639417989	0.640740741	0.562414266	0.5781893	0.615460268
Fructose 6-phosphate	3	0.00882	0.590740741	0.634920635	0.6934156	0.5325789	0.484166
Carnitine (C5) 2	3	0.04605	0.486772487	0.562433862	0.579561	0.6210562	0.5235051
3-Indolepropionic acid	3	0.01759	0.338359788	0.425925926	0.5260631	0.621056241	0.6184107
Negative mode (n = 16)							
**Pyroglutamate**	1	<0.0001	0.768656716	0.585959093	0.427983539	0.721193416	0.694090265
**Glutamate**	1	<0.0001	0.759535655	0.602542841	0.592592593	0.727709191	0.698745193
**Aspartate**	1	<0.0001	0.752072968	0.566058596	0.59122085	0.756515775	0.697632058
Inosine	1	<0.0001	0.750967385	0.383637369	0.289437586	0.80521262	0.738413277
Ribose 5-phosphate	1	<0.0001	0.717799889	0.632393588	0.679012346	0.743141289	0.706233556
Malate	1	0.00073	0.705638474	0.490878939	0.542524005	0.657407407	0.617891115
Taurine	1	<0.0001	0.701216142	0.661691542	0.691358025	0.736282579	0.705322809
Dimethylglycine	1	0.00106	0.696517413	0.565505804	0.574759945	0.679698217	0.652094718
3-Hydroxybutyric acid	1	0.02014	0.65726921	0.410724157	0.447187929	0.621399177	0.621635296
Gluconic acid	1	0.00342	0.640961857	0.524599226	0.49382716	0.687242798	0.60736693
Hippuric acid	1	0.03224	0.639579878	0.637368712	0.56515775	0.574759945	0.603825137
Histidine	1	0.02751	0.556937535	0.559977888	0.547325103	0.656378601	0.558894961
3-Indolepropionic acid	1	0.04526	0.355721393	0.426755113	0.534979424	0.610768176	0.599979761
**Lactate**	2	<0.0001	0.769209508	0.617468214	0.598765432	0.740397805	0.709674155
**Phenylalanine**	3	0.00148	0.691265893	0.53620785	0.581618656	0.586762689	0.594312892

Tryptophan	3	0.04146	0.532338308	0.592592593	0.557613169	0.595336077	0.496255819

AMP: Adenosine monophosphate, CIN1: cervical intraepithelial neoplasia 1, CIN2/3: cervical intraepithelial neoplasia 2 or 3, CX CAN: cervical cancer. [a] Kruskal-Wallis test in continuous variables and chi-square test in categorical data.

© 2019 by the authors. Licensee MDPI, Basel, Switzerland. This article is an open access article distributed under the terms and conditions of the Creative Commons Attribution (CC BY) license (http://creativecommons.org/licenses/by/4.0/).
